# Investigation of sirtuin 1 polymorphisms in relation to the risk of colorectal cancer by molecular subtype

**DOI:** 10.1038/s41598-020-60300-2

**Published:** 2020-02-25

**Authors:** Rok Hrzic, Colinda C. J. M. Simons, Leo J. Schouten, Manon van Engeland, Piet van den Brandt, Matty P. Weijenberg

**Affiliations:** 1 Department of Epidemiology, GROW – School for Oncology and Developmental Biology, Maastricht University, Maastricht, the Netherlands; 2 Department of International Health, Care and Public Health Research Institute, Maastricht University, Maastricht, the Netherlands; 3 Department of Pathology, GROW – School for Oncology and Developmental Biology, Maastricht University Medical Center+, Maastricht, the Netherlands; 4 Department of Epidemiology, CAPHRI – School for Public Health and Primary Care, Maastricht University Medical Center+, Maastricht, the Netherlands

**Keywords:** Cancer epidemiology, Cancer prevention

## Abstract

Sirtuin 1 (SIRT1), a histone deacetylase, is involved in maintenance of genetic stability, inflammation, immune response, metabolism (energy-sensing molecule) and colorectal tumorigenesis. We investigated SIRT1’s specific role in colorectal tumorigenesis by studying *SIRT1* polymorphisms in relation to colorectal cancer (CRC) risk by microsatellite instability (MSI) and CpG island methylator phenotype (CIMP) status. The Netherlands Cohort study (NLCS) was initiated in 1986 and includes 120,852 participants in a case-cohort design. CRC tumour samples were available for incident cases between 1989 and 1993. Toenail deoxyribonucleic acid (DNA) was used for genotyping of two *SIRT1* tagging variants (rs10997870 and rs12778366). Excluding the first 2.3 years of follow-up, subcohort members and CRC cases with no toenail DNA available and those with low sample call rates, and CRC cases with no tumour DNA available left 3478 subcohort members and 533 CRC cases. Cox regression was utilised to estimate hazard ratios (HRs) for MSI and CIMP positive and negative tumours by *SIRT1* genotypes. The results were that the rs12778366 TC/CC versus TT genotype was inversely associated with MSI CRC (HR = 0.41, 95% confidence interval: 0.20, 0.88), while no association was found with the risk of an MSS tumour (TC/CC versus TT carriers: HR = 1.13, 95% CI: 0.89, 1.44). No significant associations were found between other *SIRT1* genotypes and CRC subtypes. In conclusion, the results suggest a role for *SIRT1* polymorphisms in colorectal tumorigenesis, particularly MSI CRC.

## Introduction

Colorectal cancer (CRC) heterogeneity depends in part on the genetic alterations of the tumorous tissue, which are thought to reflect different CRC aetiology^1–4^. Several CRC pathways have been characterised, and these include the Microsatellite Instability (MSI) and the CpG Island Methylator Phenotype (CIMP) pathways^5^. The underlying mechanism of MSI related CRC is a defect in deoxyribonucleic acid (DNA) mismatch repair function, driven by hypermethylation of the *human MutL Homolog 1 (hMLH1)* gene. In this sense, there is overlap between the CIMP and MSI pathways, since *hMLH1* can be one of multiple methylation targets. MSI can be detected in about 15% of CRC cases, and is associated with a proximal location, older age, female sex and better disease outcome^6^. The CIMP pathway is characterized by hypermethylation of multiple genetic loci that contain CpG islands, often located in regulatory regions of genes. This in turn abrogates transcription of the affected genes. CIMP positive tumours can be detected in approximately 20% of CRC cases, although percentages vary greatly between studies, as there is a lack of consensus on the definition of CIMP^7^. CIMP tumours have similar characteristics as MSI tumours (proximal location, older age, female sex), with the exception that the majority of studies on clinical outcomes show evidence of a poor outcome, in contrast to MSI tumours^8,9^. We have previously shown that body mass index (BMI) was more strongly and significantly associated with the risk of MSI tumours as opposed to microsatellite stable (MSS) tumours^10^, while early life indicators of energy balance (BMI at 20 years of age^11^ and severe transient early life energy restriction that occurred during the Dutch Hunger Winter of 1944–45^12^ were significantly associated with the risk of CIMP positive CRC.

SIRT1, one of the seven sirtuins present in mammals, is a histone deacetylase and is involved in multiple processes including differentiation, neurodegeneration, cell death, control of gene expression, tumorigenesis and aging^13^ and has a role in metabolism as an energy-sensing molecule^14^. The precise role of SIRT1 in carcinogenesis (tumour suppressor or oncogene) remains controversial^15^. For example, there is evidence that SIRT1 acts as an oncogene via its stress-mediated anti-apoptotic activities via p53 and forkhead box O (FOXO) pathways, while an important tumour suppressor role of SIRT1 includes the promotion of DNA repair, maintaining genetic stability^16^. SIRT1’s role in maintaining genetic stability, but also its role in inflammation, which is thought to potentially underlie methylation^17^, could tie SIRT1 activity to the risk of developing an MSI or CIMP colorectal tumour in particular.

We have previously studied the association between *SIRT1* polymorphisms and CRC risk in the large population-based Netherlands Cohort Study (NLCS)^18^ after 20.3 years of follow-up. *SIRT1* variants are markers of gene involvement. We found that rs12778366 CC versus TT genotype decreased CRC and colon cancer risks in women (HR for CRC = 0.53, 95% confidence interval: 0.30–0.94) but not men using 20.3 years of follow-up, but no conclusions could be made as regards to a possible interaction between *SIRT1* variants and metabolic risk factors as the direction of associations was not always conform hypothesis nor in the same genotype stratum^19^. However, that dataset did not include information on CRC subtypes as this is only available for shorter follow-up in the NLCS. Therefore, in the present study, we aimed to extend this work by investigating the association between *SIRT1* polymorphisms and CRC by MSI and CIMP status in the NLCS using 7.3 years of follow up (excluding the first 2.3 years), where tumour DNA was available. In a supplementary analysis, we explored gene-environment (GxE) interactions on multiplicative and additive scales between *SIRT1* polymorphisms and metabolic CRC risk factors (body size, physical activity and early life energy restriction) in relation to CRC by MSI and CIMP status as was done for CRC risk overall and by subsite using the 20.3 year follow-up data. These metabolic factors are known CRC risk or protective factors and SIRT1 has a role in metabolism as described above. Evidence of GxE interactions in relation to specific CRC subtypes could further substantiate SIRT1’s role in CRC tumorigenesis. Taking into account tumour heterogeneity is of importance, because associations may be masked overall if there is heterogeneity in associations with specific CRC subtypes.

## Materials and Methods

### Population and design

The Netherlands Cohort Study on diet and cancer was initiated in September of 1986 and includes 120 852 Dutch men and women between 55 and 69 years of age at initiation. The study participants filled in a baseline questionnaire that included demographic and lifestyle factors, including information on body size, physical activity and place of residence (as a proxy for early life energy restriction)^18^. The baseline questionnaire also included a 150-item semi-quantitative food frequency questionnaire (FFQ), which was subsequently tested for validity and reproducibility. The FFQ was found to rank individuals according to food intake when compared to a dietary record and was found to be a stable indicator of food intake for at least 5 years^20,21^. DNA isolation and genotyping, processing of questionnaires and follow-up were undertaken using a case-cohort approach. A random subcohort of size n = 5000 was selected at baseline in 1986. Participants that reported cancer other than skin cancer at baseline were excluded, leaving 4774 subcohort members for follow-up for vital status to estimate the accumulated person-time at risk. Approximately 75% of the participants sent in toenail clippings at baseline, which have been used for DNA isolation and genotyping, including for determination of *SIRT1* variants.

The whole cohort was followed up for incident cancer cases via linkage to the Dutch cancer registry and the national pathology database (PALGA)^22^. This study limits itself to the follow-up period between January 1st, 1989 and December 31^st^, 1993, because tumour tissue samples have been collected for the incident CRC cases that occurred in this period. The first 2.3 years were excluded because of incomplete coverage of PALGA, the national pathology database. Exclusion of the first 2.3 years also limits the potential for preclinical disease having influenced baseline self-reported exposures. Sufficient tumour DNA was available for 732 CRC cases out of 929 and several molecular markers were determined in the isolated tumour DNA from these cases, including MSI and CIMP. Of the 4774 subcohort members at baseline, 4657 remained for follow-up in 1989. Of these subcohort members, 3719 (79.9%), and of 732 incident cases of CRC (ICD-O-3 C18–20), 557 cases (76.1%) had available toenail DNA. Toenail DNA extraction was performed using a previously validated and described method by Cline *et al*.^23^, with subsequent modifications^24^. The yield of this method was reported on in a separate report^25^. Of 3719 subcohort members and 557 incident cases of CRC, 3478 subcohort members and 533 CRC cases had a sample call rate >95% and were used in analyses.

**Figure 1:**
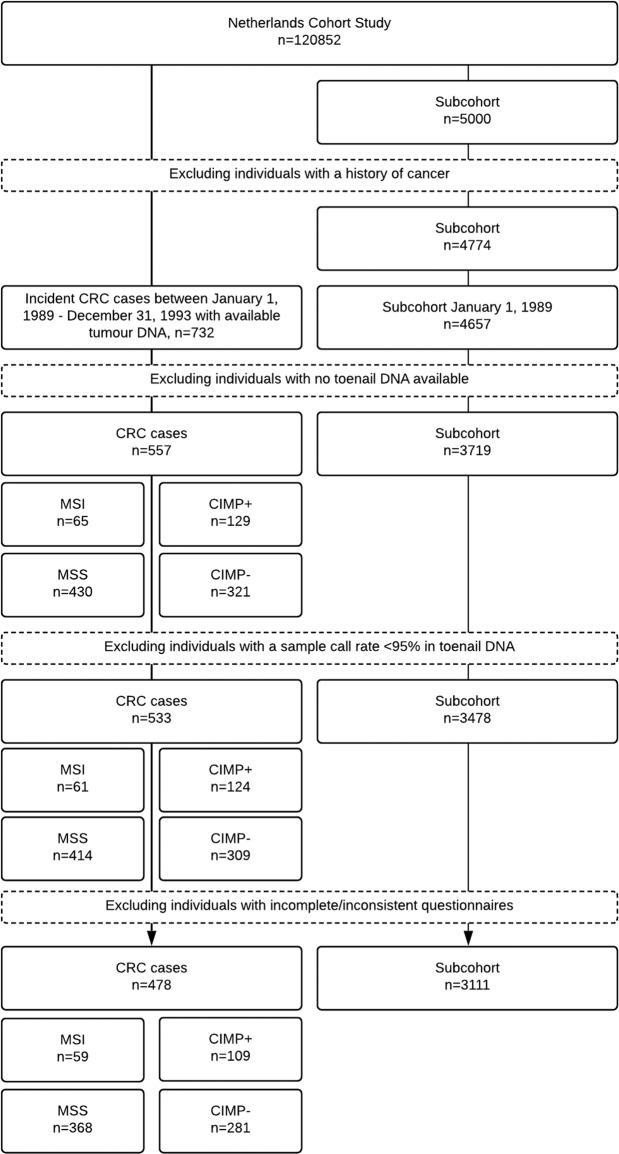
Available subcohort members, MSI and MSS, and CIMP positive and negative CRC cases in the Netherlands Cohort Study (1989–1993).

MSI was determined by a pentaplex polymerase chain reaction (PCR) using five mononucleotide repeats: BAT-26, BAT-25, NR-21, NR-22 and NR-24, where it was required that allelic size varied in more than three repeats to be classified as MSI^7,26^. CIMP was determined based on the methylation status of a panel of 5 Weisenberger markers (*RUNX3, SOCS1, NEUROG1, CACNA1G, IGF2*)^27^. Based on the number of these markers positive for methylation, CIMP status was determined as positive when 3 or more of 5 markers are present, which was tested by a methylation-specific PCR. For a number of tumour samples associated with incident cases of CRC the subtyping analysis identified them according to both the MSI/MSS as well as the CIMP +/− subtype. The number of subtyped cases in Table 1 can therefore exceed the total number of incident cases in Fig. 1.

**Table 1:** Baseline characteristics of subcohort members and CRC cases from the Netherlands Cohort Study (1989–1993).

	Subcohort members (N = 3111)		Colorectal cancer cases by MSI status				Colorectal cancer cases by CIMP status			
			MSI (N = 59)		MSS (N = 368)		CIMP + (N = 109)		CIMP- (N = 281)	
	N (%)	Mean (SD)	N (%)	Mean (SD)	N (%)	Mean (SD)	N (%)	Mean (SD)	N (%)	Mean (SD)
Age (years)		61.3 (4.2)		63.2 (4.7)		62.8 (4.1)		62.9 (4.2)		62.6 (4.2)
Sex (male)	1623 (52.2)		33 (55.9)		215 (58.4)		65 (59.6)		165 (58.7)	
rs10997870										
TT	1,232 (39.6)		23 (39.0)		139 (37.8)		44 (40.4)		102 (36.3)	
TG	1,464 (47.1)		33 (56.0)		168 (45.7)		48 (44.0)		134 (47.7)	
GG	415 (13.3)		3 (5.1)		61 (16.6)		17 (15.6)		45 (16.0)	
rs12778366										
TT	2250 (72.3)		51 (86.4)		258 (70.1)		84 (77.1)		199 (70.8)	
TC	790 (25.4)		7 (11.9)		102 (27.7)		20 (18.4)		77 (27.4)	
CC	71 (2.3)		1 (1.7)		8 (2.2)		5 (4.6)		5 (1.8)	
BMI, kg/m^2^		25.0 (3.1)		25.4 (2.7)		25.4 (3.2)		25.7 (2.7)		25.4 (3.3)
Waist circumference, median split (Dutch clothing sizes) (above median)^b^	1731 (58.8)		35 (66.0)		231 (66.2)		70 (70.0)		173 (65.0)	
Height, cm		171.1 (8.6)		172.2 (7.7)		172.5 (8.4)		172.1 (7.8)		172.6 (8.1)
BMI at age 20, kg/m^2^		21.6 (2.6)		21.7 (2.6)		21.7 (2.4)		22.1 (2.4)		21.6 (2.4)
Physical activity (low)^a^	1,211 (41.5)		24 (42.1)		174 (49.2)		54 (52.4)		125 (46.0)	
Energy restriction during Dutch Hunger Winter (exposed)^b^	724 (23.3)		10 (17.0)		75 (20.4)		19 (17.4)		60 (21.4)	
Family history of CRC (yes)	183 (5.9)		4 (6.8)		47 (12.8)		11 (10.1)		32 (11.4)	
**Smoking**										
Never	1069 (34.4)		18 (30.5)		129 (35.1)		37 (33.9)		92 (32.7)	
Ex	1187 (38.2)		26 (44.1)		169 (45.9)		50 (45.9)		129 (45.9)	
Current	855 (27.5)		15 (25.4)		70 (19.0)		22 (20.2)		60 (21.4)	
Alcohol consumption (≥30 g/d)	289 (9.4)		5 (8.5)		43 (11.9)		15 (13.8)		31 (11.2)	
Total daily calorie intake (kcal/day)		1393.5 (507.6)		1972.1 (506.1)		1940.6 (466.8)		1931.4 (450.0)		1949.3 (487.5)
Red meat consumption (g/day)		64.1 (35.2)		59.6 (29.9)		63.4 (34.1)		63.6 (31.7)		62.0 (32.9)
Fruit, vegetables, and grain consumption (g/day)		383.7 (161.1)		373.1 (197.9)		391.4 (165.6)		404.6 (167.1)		374.8 (160.0)

Abbreviations: MSI, microsatellite instability; CIMP, CpG island methylator phenotype; N, number of; SD, standard deviation.

^a^Low versus moderate or high physical activity was <8 versus ≥8 kJ/min of occupational energy expenditure in men and ≤30 versus>30 min/day of non-occupational physical activity in women.

^b^Exposure status was based on the place of residence during the Dutch Hunger Winter, i.e. lived in a Western city versus lived in a Western rural area or non-western area.

All methods were carried out in accordance with relevant guidelines and regulations and all subjects gave informed consent by returning the baseline questionnaire. The NLCS has been approved by the institutional review boards of the TNO Quality of Life Research Institute (Zeist, the Netherlands; in Dutch known as the Nederlandse Organisatie voor Toegepast **N**atuurwetenschappelijk **O**nderzoek (TNO)) and Maastricht University (Maastricht, the Netherlands).

### Exposure assessment and main covariates

Two tagging single nucleotide polymorphisms (SNPs), rs10997870 (T/G) and rs12778366 (T/C), were selected using aggressive tagging, which covered 100% of the genetic variation in *SIRT1* (±5 kb) at a minor allele frequency of 5% or higher using Haploview (version 4.2, Broad Institute, Cambridge, MA) with the HapMap Caucasian European (CEU) population (Utah Residents with Northern and Western European Ancestry) and keeping default settings (which included an r^2^ threshold of 0.8). SNP and sample call rates were near perfect and Hardy-Weinberg equilibrium was not violated.

Body size information was derived from the baseline questionnaire and includes data on height in cm and weight in kg at baseline and at age 20 to derive body mass index in kg/m^2^ and trouser/skirt size (Dutch sizes) as a proxy for waist circumference. In previous studies, trouser/skirt size was found to be a useful proxy for waist circumference in weight-stable subcohort members, and was associated with other cancer risk as expected for waist circumference^28^. Similarly, several studies have found self-reported information on weight and height to be reliable compared to direct measurement in various adult populations^29–31^. The aim was to approximate abdominal fatness by using trouser/skirt size adjusted for BMI. Trouser/skirt size was categorized as lower, or equal or greater to median sex-specific size, with the above median individuals considered being at greater risk. BMI was similarly analysed utilising a median split, but was included as a continuous variable when considered a covariate.

Physical activity level was derived from baseline questionnaire information on occupational history in men and non-occupational physical activity in women. Using a rating system developed by Hettinger *et al*.^32^, occupational energy expenditure (<8, 8–12, >12 kJ/min) was calculated based on the nature of occupation for the longest held job. In women, non-occupational physical activity was used (≤30, >30–60, >60–90, >90 min/day), as most of the women in the subcohort identified as homemakers exclusively or held jobs only briefly and in the distant past^7^. Non-occupational physical activity was a sum measure of daily walking/cycling (min/day) and recreational walking/cycling, gardening/doing odd jobs and sports/gymnastics (never, <1, 1–2, >2 hours/week). Categories from both sexes were combined into two categories, low (<8 kJ/min for men and ≤30 min/day for women) and moderate or high levels of physical activity (≥8 kJ/min for men and ≥30 min/day for women), where the low physical activity category was considered to include individuals at higher risk.

Lastly, early life energy restriction was determined by the information on the place of residence during the Hunger Winter in 1944–45 on the questionnaire, where residence in a Western city was considered an indicator of severe early life energy restriction (daily rations of ~400–800 kcal/day). The validity of this proxy variable for early life energy restriction has been indicated by its ability to predict a delayed menarche contrary to secular trend patterns in the region^33^, its ability to explain a disruption in the expected increasing trend in birth weights with increasing birth order^34^ and via a follow-up questionnaire completed by female subcohort members from the present study several years after baseline, which showed a high level of concordance between residence in a Western city during the Hunger Winter and the experience of severe hunger^35^.

### Statistics

Cox proportional hazards regression was utilized to estimate hazard ratios (HRs) and 95% confidence intervals (CIs) for the association between *SIRT1* genetic variation (rs10997870 and rs12778366) and the risk of CRC by MSI and CIMP status. The event of interest was the first diagnosis of CRC. A common dominant model of risk was assumed for the SNPs to preserve power. Potential confounding by age and sex was considered when constructing the model for the primary outcome (association between *SIRT1* genotype and CRC by MSI and CIMP status), as it is unlikely that other variables could influence germline genotype and the aim was to maintain as much power as possible for the analysis.

Tests for heterogeneity were performed to evaluate differences between tumour subtypes (MSI versus MSS and CIMP + versus CIMP-) using a competing risks procedure. P-values were calculated based on a bootstrapping method that was developed for the case-cohort design. This procedure has been described in detail elsewhere^36,37^.

GxE interactions were explored by comparing individuals with higher versus lowest expected risk by computing composite categories of body composition, physical activity and energy restriction, and *SIRT1* variants. While the reference level of the *SIRT1* variants (low risk allele) is unknown, the reference (low risk) levels of the other exposures are known and have been discussed above. The default assumption was that the homozygous wild-type allele is the low risk category. Interaction on the additive scale was explored by calculating the relative excess risk due to interaction (RERI). To accomplish this, the category of lowest risk first had to be identified by calculating HRs for 2×2 combinations of genotype and body size variables (e.g. rs12778366 genotype by waist circumference using the median split), with the data then appropriately recoded so that the category with the lowest risk of CRC became the reference. While this approach could lead to reference categories in opposition to current understanding of cancer risk, it is described in the literature as the optimal approach^38^. The RERI was derived from the formula RERI = RR_11_ – RR_10_ – RR_01_ + 1, where RR denotes relative risk and 1 and 0 denote comparison versus reference categories, with the position of this number denoting the first and second exposure of two combined exposures^39^. Corresponding 95% bias-corrected confidence intervals were estimated by bootstrapping (n bootstrap samples = 1000)^40^. Considering the explorative nature of this analysis, we did not correct for multiple testing. We also tested for the presence of interaction on the multiplicative scale using the Wald test. In the case of a significant multiplicative interaction, a subsequent stratified analysis was performed. The models for composite categories included different sets of potential confounders, depending on the specific exposures combined in the composite exposure. This way, three multivariable-adjusted models were used. First, the composite *SIRT1* genotype and body composition models [separate models for waist circumference (trouser/skirt size adjusted for BMI (kg/m^2^)) and BMI and BMI at 20 (median split categories)] included age, sex, physical activity (median split), diet [total energy intake (kcal/day), alcohol intake (binary split, ≥30 g/day), consumption of red meat, and consumption of vegetables and grains (g/day)], first-degree family history of CRC (yes/no) and smoking status (never, ex, current) as potential confounders. Second, the composite *SIRT1* genotype and physical activity (occupational for men and non-occupational for women) model included age, sex, BMI, diet (as above), first-degree family history of CRC and smoking status as potential confounders. Last, the composite *SIRT1* genotype and energy restriction model included age, sex, BMI, physical activity, diet (as above), first-degree family history of CRC and smoking as potential confounders.

Data analysis was performed with Stata, version 13 (StataCorp LP, College Station, Texas). For all analyses, a two-sided Type I error probability of 0.05 has been used as the cut off value. The proportional hazards assumption was tested via the scaled Schoenfeld residuals and by a visual inspection of the minus-log-log-transformed hazard curves. Standard errors were estimated using the robust Huber-White sandwich estimator to account for the additional variance introduced by sampling the subcohort from the entire cohort^41^.

## Results

The flow of MSI and MSS cases and CIMP positive and negative cases and subcohort members with their respective numbers at points of exclusion, irrespective of missing values in potential confounders, is outlined in Fig. 1. Table 1 displays the baseline characteristics of the population under study for subcohort members and CRC cases by MSI and CIMP status. CRC cases, in particular MSI cases, were, on average, older than the subcohort members and slightly more often male. In terms of the genetic variants under investigation, all groups were fairly similar in distribution across rs10997870 genotypes, except for the MSI group, but, then again, the number of MSI cases was low. The distribution of subcohort members, MSS cases and CIMP-negative cases across rs12778366 genotypes appeared to differ from that observed for MSI and CIMP-positive cases. CRC cases differed from subcohort members in terms of height, BMI, waist circumference, physical activity level, BMI at age 20 (CIMP-positive cases) and prevalence of early life energy restriction in a manner consistent with knowledge on these factors as being risk or protective factors, albeit in particular subgroups and in some cases only slightly. CRC subgroups were more likely to have a family history of CRC and differed from the subcohort in their smoking habits (i.e. they were more often ex-smokers) and alcohol intake (i.e. all groups, except MSI cases, were more often heavy drinkers). Groups differed less with respect to intake of red meat and fruit, vegetables and grains.

Table 2 displays the results of the main analysis of the association between the two *SIRT1* tagSNPs rs10997870 and rs12778366, assuming a dominant inheritance mode and the risk of CRC by MSI and CIMP status. Rs10997870 was not associated with any of the CRC subtypes considered. Rs12778366 was not associated with CRC in the context of the CIMP subtype (CIMP positive and negative tumours), but there was a significant inverse association between rs12778366 and the risk of developing an MSI tumour (TC/CC versus TT carriers: HR = 0.41, 95% CI: 0.20, 0.88). In contrast, rs12778366 was not associated with the risk of an MSS tumour (TC/CC versus TT carriers: HR = 1.13, 95% CI: 0.89, 1.44). All heterogeneity tests were not statistically significant.

**Table 2:** Association of *SIRT1* variants with the risk of CRC by MSI and CIMP status in the Netherlands Cohort Study (1989–1993).

	MSI CRC			MSS CRC			
	N cases / PY	HR	(95% CI)	N cases / PY	HR	(95% CI)	Heterogeneity test (p-value)
rs10997870 TT	23 / 5987	1	Ref.	139 / 6040	1	(Ref.)	
rs10997870 TG/GG	36 / 9102	1.04	(0.61, 1.76)	229 / 9189	1.09	(0.88, 1.37)	0.863
	CIMP + CRC			CIMP- CRC			
	N cases / PY	HR	(95% CI)	N cases / PY	HR	(95% CI)	Heterogeneity test (p-value)
rs10997870 TT	44 / 5987	1	(Ref.)	102 / 6088	1	(Ref.)	
rs10997870 TG/GG	65 / 9102	0.98	(0.67, 1.45)	179 / 9245	1.17	(0.91, 1.51)	0.438
	MSI CRC			MSS CRC			
	N cases / PY	HR	(95% CI)	N cases / PY	HR	(95% CI)	Heterogeneity test (p-value)
rs12778366 TT	51 / 10916	1	(Ref.)	258 / 11027	1	(Ref.)	
rs12778366 TC/CC	8 / 4173	0.41	(0.20, 0.88)	110 / 4201	1.13	(0.90, 1.44)	0.161
	CIMP + CRC			CIMP- CRC			
	N cases / PY	HR	(95% CI)	N cases / PY	HR	(95% CI)	Heterogeneity test (p-value)
rs12778366 TT	84 / 10916	1	(Ref.)	199 / 11110	1	(Ref.)	
rs12778366 TC/CC	25 / 4173	0.79	(0.50, 1.24)	82 / 4223	1.10	(0.84, 1.44)	0.209

Abbreviations: CRC, colorectal cancer; CIMP, CpG island methylator phenotype; CI, confidence interval; HR, hazard ratio; MSI, microsatellite instability; MSS, microsatellite stable; N, number of; PY, person-years at risk; Ref., reference.

*Hazard ratios were adjusted for age (years) and sex.

We explored interactions on both additive and multiplicative scales between *SIRT1* rs10997870 and rs12778366 genotypes (dominant model), and six metabolic CRC risk factors recoded into a binary fashion for reasons of power (height, adult BMI, trouser/skirt size, BMI at age 20, physical activity, and early life energy restriction using the place of residence during the Dutch Hunger Winter as a proxy variable). On the additive scale, as shown in Supplemental Table 1, the RERIs estimated based on composite SNP-risk factor categories were not found to be significant. No significant interactions were discovered on the multiplicative scale and so we refrained from performing further stratified analyses considering the explorative nature of these GxE interaction analyses (Supplemental Table 1).

## Discussion

In the present investigation, the association between germline genetic variation in *SIRT1* and CRC by MSI and CIMP status was examined. The TC and CC genotypes of the rs12778366 SNP were found to be significantly inversely associated with the risk of developing an MSI subtype CRC as compared to the TT genotype, while there was no association between rs12778366 and the risk of an MSS tumour or CRC by CIMP status. There was no statistically significant heterogeneity in the association between the genotype and the MSI and MSS tumor subtypes. The TG and GG genotypes compared to the TT genotype of the rs10997870 SNP in relation to CRC by molecular subtype did not yield significant results. Exploratory gene-environment interaction analyses, showed no interactions on the multiplicative and the additive scale between *SIRT1* variations and known metabolic risk factors of CRC in determining the risk of incident CRC by molecular status.

To the best of our knowledge, no previous studies examined the influence of *SIRT1* genotype on CRC risk by MSI and CIMP status. However, research has been performed on the association of *SIRT1* polymorphisms and risk of other cancers. A recent study of Egyptian women found a positive association between *SIRT1* rs12778366 TT genotype and the risk of breast cancer as compared to TC and CC genotypes, and carrying the rs12778366 TT genotype was associated with higher serum SIRT1 levels compared to TC and CC genotypes in breast cancer cases but not controls, while, overall, breast cancer cases had significantly higher serum SIRT1 levels than controls^42^. A study of pituitary adenoma patients found that the CC *SIRT1* rs12778366 genotype was more common in patients compared to healthy controls, while the TC genotype was rarer in patients and no difference between the groups was found when considering the TT genotype^43^. The key finding of the present study was the significant inverse association between MSI CRC and the rs12778366 SNP TC or CC genotype, a SNP-cancer pattern apparently consistent with that found in the study on breast cancer risk.

In relation to specifically MSI and CIMP in CRC, two previous studies investigated SIRT1 expression and one found that SIRT1 overexpression was more common in tumour tissue samples of both MSI- and CIMP-high colon cancers than other MSI/CIMP subtypes^44^, while the other found that loss of SIRT1 expression was associated with MSI-high CRC^45^. We do not know whether the decreased risk of developing an MSI tumour associated with *SIRT1* rs12778366 CC and TC genotypes, as found in this study, corresponded to higher or lower levels of SIRT1 expression in colorectal cells or serum, since we did not measure SIRT1 expression. The findings of the study on breast cancer indicated higher serum SIRT1 expression levels to be associated with the rs12778366 TT genotype in breast cancer cases, which was also associated with an increased risk of breast cancer, suggesting a tumour promoting role of SIRT1^42^. The nature of the relationship between *SIRT1* genotypes and SIRT1 expression and between SIRT1 expression and CRC risk remains to be elucidated and may be complex. For example, recent results in mouse models suggested dose-dependent effects of SIRT1 expression levels on cancer development, i.e. intestine-specific heterozygous deletion of Sirt1 in mice resulted in enhanced tumour formation by enhancing glutamine metabolism, while homozygous deletion of Sirt1 in mice showed reduced cancer development by triggering cellular apoptotic pathways^46^.

In evaluating the main finding of this study, one must remain cautious, especially in the light of a positive, albeit non-significant association between the same *SIRT1* SNP and CIMP positive CRC, given that MSI and CIMP pathways often overlap. However, the overlap between the two pathways is incomplete and the size of association discovered quite substantial (HR = 0.41), whereas the lack of significant associations in other combinations may be just as likely due to a lack of power of this study to detect them as due to them not existing in nature. Nevertheless, despite the absence of an association between *SIRT1* SNPs and CIMP in CRC in this study, there is substantial biological plausibility for an association between *SIRT1* SNPs and MSI in CRC specifically. This is not only considering SIRT1’s role in maintaining genetic stability, but also considering that *SIRT1* transcription, for example, has been found to be persistently reduced in tissues during chronic inflammation and activation of the immune response^14^, including chronic bowel inflammation, where *SIRT1* activators are investigated as potential treatments *in vitro* and in animal models^47,48^. One of the characteristics of MSI CRC is the pathological finding of immune cell invasion of the tumour and an upregulation of various immune inhibitory molecules, pointing to a possible key role of the immune system in MSI colorectal tumorigenesis^49^. Furthermore, as already mentioned in the introduction, an analysis using NLCS data from 20.3 years follow-up showed that rs12778366 CC versus TT genotype decreased CRC and colon cancer risks in women but not men^19^. This analysis was well-powered and is consistent with the current result in that MSI tumors more often have a proximal than distal location and more often occur in women than men.

Turning to the GxE interactions portion of this analysis, no significant interactions were found, which could be true in nature or due to the limited statistical power in these analyses, especially when considering MSI and CIMP+ as endpoints. While information on tumour molecular subtype was lacking in the previous study performed within the NLCS using 20.3 years of follow-up, the number of CRC cases in that study was much higher than in the current study. However, we could not make conclusions as regards to a possible interaction in that study either as described in the introduction^19^. Biologically speaking, SIRT1 acts as an energy-sensing molecule^14^ and an increase in SIRT1 activity occurs during nutrient deficiency and exercise in mammalian cells^13,50,51^, and effects of SIRT1 have been postulated as to potentially partially explain the association between calorie restriction and a reduction in cancer risk^50^. An interaction between SIRT1 and metabolic CRC risk factors was therefore hypothesized. Collectively, however, there is no support for this hypothesis when considering current and previous work in the NLCS^19^ in which *SIRT1* rs12778366 and rs10997870 were used as markers of gene involvement.

The strengths of this study include its prospective design and a substantial follow-up period, which resulted in a significant number of unselected cancer cases with available tumour material. This is also one of the few studies with information on both MSI and CIMP CRC status as measured in tumour tissue, as well as information on germline *SIRT1* genotype variation. A key weakness is the absence of expression data and the limited power, especially in certain combined subcategories of exposure, which could have been responsible for the probable lack of power in the GxE portion of the analysis, although power also depends on the size of a possible interaction effect. The limited power was mainly due to a drop in numbers due to incomplete overlap between unsuccessful molecular analyses in tumour tissue and toenail DNA. Missing data, however, were likely missing at random and, therefore, the incident CRC cases identified within the NLCS, which has nationwide coverage, most likely remain an unselected case group.

In conclusion, the results suggest a role for *SIRT1* polymorphisms in colorectal tumorigenesis, particularly MSI CRC.

## Supplementary information

Supplemental Table 1.
