# Supplementary material for: Investigation of sirtuin 1 polymorphisms in relation to the risk of colorectal cancer by molecular subtype
Source: Sci Rep. 2020 Feb 25;10:3359. doi: 10.1038/s41598-020-60300-2 (PMC7042277; doi:10.1038/s41598-020-60300-2)
Supplement: Supplementary file 1 — Supplemental Table 1. [file 41598_2020_60300_MOESM1_ESM.docx]

**Investigation of sirtuin 1 polymorphisms in relation to the risk of colorectal cancer by molecular subtype**

Running title Sirtuin 1 and the risk of colorectal cancer by molecular subtype

Rok Hrzic^1,2,†^, Colinda C.J.M. Simons^1,†^, Leo J. Schouten^1^, Manon van Engeland^3^, Piet van den Brandt^1,4^, Matty P. Weijenberg^1,*^

^1^ Department of Epidemiology, GROW – School for Oncology and Developmental Biology, Maastricht University, Maastricht, the Netherlands

^2^ Department of International Health, Care and Public Health Research Institute, Maastricht University, Maastricht, the Netherlands

^3^ Department of Pathology, GROW – School for Oncology and Developmental Biology, Maastricht University Medical Center+, Maastricht, the Netherlands

^4^ Department of Epidemiology, CAPHRI- School for Public Health and Primary Care, Maastricht University Medical Center+, Maastricht, the Netherlands

^†^ Joint first authorship - these authors contributed equally to this work.

**Keywords** sirtuin 1, metabolic risk factors, colorectal carcinoma, microsatellite instability phenotype, CpG island methylator phenotype

**Funding** This work was supported by the World Cancer Research Fund (grant number 2013/673 to MPW); and the Health Foundation Limburg (grant to MPW).

**^*^** Correspondence and request for reprints should be addressed to: Matty P. Weijenberg, Department of Epidemiology, GROW – School for Oncology and Developmental Biology, Maastricht University, P.O. Box 616, 6200 MD Maastricht, the Netherlands, Phone: +31 43 3882358, Telefax: +31 43 3884128, E-mail: [mp.weijenberg@maastrichtuniversity.nl](mailto:mp.weijenberg@maastrichtuniversity.nl)

| **Supplemental Table 1.** Gene-environment interactions on the multiplicative and additive scales between *SIRT1* genotypes and physical activity and early life energy restriction during the Dutch Hunger Winter in relation to the risk of MSI or CIMP CRC subtypes in the Netherlands Cohort Study (1989-1993) | | | | | | | | | | | |
| --- | --- | --- | --- | --- | --- | --- | --- | --- | --- | --- | --- |
| **Outcome** | **Model** |  | | | | | | | | | |
| MSI CRC |  | rs10997870 TT | | | | rs10997870 TG or GG | | |  |  |  |
|  |  | N cases / PY | HR | (95% CI) | N cases / PY | | HR | (95% CI) | Multiplicative interaction  (p-value) | RERI | (95% CI) |
|  | BMI (below median)^c^ | 10 / 3068 | 1.00 | (0.43, 2.37) | 16 / 4702 | | 1 | (Ref.) |  |  |  |
|  | BMI (above median) | 12 / 2775 | 1.26 | (0.50, 3.16) | 18 / 4130 | | 1.20 | (0.54, 2.65) | 0.94 | 0.06 | (-1.54, 1.65) |
|  |  | rs12778366 TT | | | | rs12778366 TC or CC | | |  |  |  |
|  |  | N cases / PY | HR | (95% CI) | N cases / PY | | HR | (95% CI) | Multiplicative interaction  (p-value) | RERI | (95% CI) |
|  | BMI (below median)^c^ | 21 / 5606 |  |  | 5 / 2164 | |  |  |  |  |  |
|  | BMI (above median) | 27 / 5044 |  |  | 3 / 1860 | | data not shown due to <5 cases | | data not shown due to <5 cases |  | DNC |
|  |  | rs10997870 TT | | | | rs10997870 TG or GG | | |  |  |  |
|  |  | N cases / PY | HR | (95% CI) | N cases / PY | | HR | (95% CI) | Multiplicative interaction  (p-value) | RERI | (95% CI) |
|  | BMI at 20 (below median)^c^ | 11 / 2559 | 1.87 | (0.77, 4.56) | 9 / 3842 | | 1 | (Ref.) |  |  |  |
|  | BMI at 20 (above median) | 8 / 2416 | 1.28 | (0.46, 3.56) | 21 / 3800 | | 2.06 | (0.92, 4.61) | 0.09 | -1.65 | (-5.66, 2.36) |
|  |  | rs12778366 TT | | | | rs12778366 TC or CC | | |  |  |  |
|  |  | N cases / PY | HR | (95% CI) | N cases / PY | | HR | (95% CI) | Multiplicative interaction  (p-value) | RERI | (95% CI) |
|  | BMI at 20 (below median)^c^ | 17 / 4569 |  |  | 3 / 1833 | |  | |  |  |  |
|  | BMI at 20 (above median) | 25 / 4539 |  |  | 4 / 1677 | | data not shown due to <5 cases | | data not shown due to <5 cases |  | DNC |
|  |  | rs10997870 TT | | | | rs10997870 TG or GG | | |  |  |  |
|  |  | N cases / PY | HR | (95% CI) | N cases / PY | | HR | (95% CI) | Multiplicative interaction  (p-value) | RERI | (95% CI) |
|  | Waist circumference (below median)^c^ | 8 / 2330 | 1.07 | (0.38, 3.05) | 10 / 3573 | | 1 | (Ref.) |  |  |  |
|  | Waist circumference (above median) | 14 / 3356 | 1.30 | (0.48, 3.53) | 21 / 5029 | | 1.11 | (0.42, 2.88) | 0.89 | 0.13 | (-1.74, 1.99) |
|  |  | rs12778366 TT | | | | rs12778366 TC or CC | | |  |  |  |
|  |  | N cases / PY | HR | (95% CI) | N cases / PY | | HR | (95% CI) | Multiplicative interaction  (p-value) | RERI | (95% CI) |
|  | Waist circumference (below median)^c^ | 14 / 4210 |  |  | 4 / 1693 | |  |  |  |  |  |
|  | Waist circumference (above median) | 31 / 6133 |  |  | 4 / 2251 | | data not shown due to <5 cases | | data not shown due to <5 cases |  | DNC |
|  |  | rs10997870 TT | | | | rs10997870 TG or GG | | |  |  |  |
|  |  | N cases / PY | HR | (95% CI) | N cases / PY | | HR | (95% CI) | Multiplicative interaction  (p-value) | RERI | (95% CI) |
|  | Physical activity^a^ (medium or high)^d^ | 13 / 3367 | 1.00 | (0.43, 2.30) | 20 / 4922 | | 1.08 | (0.52, 2.23) |  |  |  |
|  | Physical activity^a^ (low) | 9 / 2227 | 1.06 | (0.45, 2.53) | 15 / 3638 | | 1 | (Ref.) | 0.81 | -0.14 | (-1.57, 1.29) |
|  |  | rs12778366 TT | | | | rs12778366 TC or CC | | |  |  |  |
|  |  | N cases / PY | HR | (95% CI) | N cases / PY | | HR | (95% CI) | Multiplicative interaction  (p-value) | RERI | (95% CI) |
|  | Physical activity^a^ (medium or high)^d^ | 29 / 6071 |  |  | 4 / 2218 | |  | |  |  |  |
|  | Physical activity^a^ (low) | 20 / 4160 |  |  | 4 / 1705 | | data not shown due to <5 cases | | data not shown due to <5 cases |  | DNC |
|  |  | rs10997870 TT | | | | rs10997870 TG or GG | | |  |  |  |
|  |  | N cases / PY | HR | (95% CI) | N cases / PY | | HR | (95% CI) | Multiplicative interaction  (p-value) | RERI | (95% CI) |
|  | Early life energy restriction^b^ (yes)^e^ | 0 / 1465 |  |  | 10 / 2079 | |  |  |  |  |  |
|  | Early life energy restriction^b^ (no) | 23 / 4522 |  |  | 26 / 7023 | | data not shown due to <5 cases | | data not shown due to <5 cases |  | DNC |
|  |  | rs12778366 TT | | | | rs12778366 TC or CC | | |  |  |  |
|  |  | N cases / PY | HR | (95% CI) | N cases / PY | | HR | (95% CI) | Multiplicative interaction  (p-value) | RERI | (95% CI) |
|  | Early life energy restriction^b^ (yes)^e^ | 8 / 2546 |  |  | 2 / 998 | |  | |  |  |  |
|  | Early life energy restriction^b^ (no) | 43 / 8370 |  |  | 6 / 3175 | | data not shown due to <5 cases | | data not shown due to <5 cases |  | DNC |
| MSS CRC |  | rs10997870 TT | | | | rs10997870 TG or GG | | |  |  |  |
|  |  | N cases / PY | HR | (95% CI) | N cases / PY | | HR | (95% CI) | Multiplicative interaction  (p-value) | RERI | (95% CI) |
|  | BMI (below median)^c^ | 56 / 3082 | 1 | (Ref.) | 102 / 4744 | | 1.32 | (0.90, 1.94) |  |  |  |
|  | BMI (above median) | 80 / 2814 | 1.67 | (1.10, 2.53) | 122 / 4171 | | 1.48 | (1.00, 2.19) | 0.13 | -0.51 | (-1.35, 0.33) |
|  |  | rs12778366 TT | | | | rs12778366 TC or CC | | |  |  |  |
|  |  | N cases / PY | HR | (95% CI) | N cases / PY | | HR | (95% CI) | Multiplicative interaction  (p-value) | RERI | (95% CI) |
|  | BMI (below median)^c^ | 109 / 5646 | 1 | (Ref.) | 40 / 2179 | | 1.10 | (0.73, 1.64) |  |  |  |
|  | BMI (above median) | 145 / 5111 | 1.36 | (1.00, 1.84) | 57 / 1874 | | 1.26 | (0.85, 1.87) | 0.55 | -0.20 | (-0.85, 0.46) |
|  |  | rs10997870 TT | | | | rs10997870 TG or GG | | |  |  |  |
|  |  | N cases / PY | HR | (95% CI) | N cases / PY | | HR | (95% CI) | Multiplicative interaction  (p-value) | RERI | (95% CI) |
|  | BMI at 20 (below median)^c^ | 65 / 2574 | 1.26 | (0.83, 1.90) | 97 / 3862 | | 1.23 | (0.84, 1.79) |  |  |  |
|  | BMI at 20 (above median) | 53 / 2449 | 1 | (Ref.) | 98 / 3840 | | 1.15 | (0.80, 1.67) | 0.53 | -0.18 | (-0.81, 0.45) |
|  |  | rs12778366 TT | | | | rs12778366 TC or CC | | |  |  |  |
|  |  | N cases / PY | HR | (95% CI) | N cases / PY | | HR | (95% CI) | Multiplicative interaction  (p-value) | RERI | (95% CI) |
|  | BMI at 20 (below median)^c^ | 111 / 4595 | 1.17 | (0.77, 1.78) | 51 / 1841 | | 1.26 | (0.79, 2.03) |  |  |  |
|  | BMI at 20 (above median) | 111 / 4598 | 1.08 | (0.72, 1.62) | 40 / 1691 | | 1 | (Ref.) | 0.59 | -0.17 | (-0.94, 0.60) |
|  |  | rs10997870 TT | | | | rs10997870 TG or GG | | |  |  |  |
|  |  | N cases / PY | HR | (95% CI) | N cases / PY | | HR | (95% CI) | Multiplicative interaction  (p-value) | RERI | (95% CI) |
|  | Waist circumference (below median)^c^ | 39 / 2348 | 1 | (Ref.) | 79 / 3600 | | 1.49 | (0.95, 2.34) |  |  |  |
|  | Waist circumference (above median) | 91 / 3386 | 1.52 | (0.94, 2.44) | 140 / 5082 | | 1.39 | (0.88, 2.20) | 0.08 | -0.62 | (-1.54, 0.31) |
|  |  | rs12778366 TT | | | | rs12778366 TC or CC | | |  |  |  |
|  |  | N cases / PY | HR | (95% CI) | N cases / PY | | HR | (95% CI) | Multiplicative interaction  (p-value) | RERI | (95% CI) |
|  | Waist circumference (below median)^c^ | 82 / 4245 | 1 | (Ref.) | 36 / 1703 | | 1.06 | (0.67, 1.67) |  |  |  |
|  | Waist circumference (above median) | 163 / 6198 | 1.13 | (0.79, 1.62) | 68 / 2270 | | 1.13 | (0.73, 1.74) | 0.84 | -0.06 | (-0.73, 0.60) |
|  |  | rs10997870 TT | | | | rs10997870 TG or GG | | |  |  |  |
|  |  | N cases / PY | HR | (95% CI) | N cases / PY | | HR | (95% CI) | Multiplicative interaction  (p-value) | RERI | (95% CI) |
|  | Physical activity^a^ (medium or high)^d^ | 69 / 3381 | 1 | (Ref.) | 111 / 4973 | | 1.11 | (0.80, 1.54) |  |  |  |
|  | Physical activity^a^ (low) | 66 / 2259 | 1.29 | (0.88, 1.88) | 108 / 3669 | | 1.31 | (0.92, 1.85) | 0.71 | -0.09 | (-0.66, 0.50) |
|  |  | rs12778366 TT | | | | rs12778366 TC or CC | | |  |  |  |
|  |  | N cases / PY | HR | (95% CI) | N cases / PY | | HR | (95% CI) | Multiplicative interaction  (p-value) | RERI | (95% CI) |
|  | Physical activity^a^ (medium or high)^d^ | 124 / 6118 | 1 | (Ref.) | 56 / 2235 | | 1.26 | (0.89, 1.79) |  |  |  |
|  | Physical activity^a^ (low) | 128 / 4217 | 1.35 | (1.01, 1.80) | 46 / 1712 | | 1.17 | (0.79, 1.72) | 0.15 | -0.45 | (-1.11, 0.21) |
|  |  | rs10997870 TT | | | | rs10997870 TG or GG | | |  |  |  |
|  |  | N cases / PY | HR | (95% CI) | N cases / PY | | HR | (95% CI) | Multiplicative interaction  (p-value) | RERI | (95% CI) |
|  | Early life energy restriction^b^ (yes)^e^ | 27 / 1479 | 1 | (Ref.) | 48 / 2101 | | 1.23 | (0.73, 2.19) |  |  |  |
|  | Early life energy restriction^b^ (no) | 112 / 4561 | 1.37 | (0.86, 2.19) | 181 / 7088 | | 1.39 | (0.89, 2.18) | 0.25 | -0.21 | (-1.01, 0.58) |
|  |  | rs12778366 TT | | | | rs12778366 TC or CC | | |  |  |  |
|  |  | N cases / PY | HR | (95% CI) | N cases / PY | | HR | (95% CI) | Multiplicative interaction  (p-value) | RERI | (95% CI) |
|  | Early life energy restriction^b^ (yes)^e^ | 51 / 2570 | 1 | (Ref.) | 24 / 1010 | | 1.25 | (0.73, 2.14) |  |  |  |
|  | Early life energy restriction^b^ (no) | 207 / 8458 | 1.30 | (0.92, 1.83) | 86 / 3191 | | 1.30 | (0.88, 1.92) | 0.47 | -0.25 | (-1.06, 0.56) |
| CIMP+ CRC |  | rs10997870 TT | | | | rs10997870 TG or GG | | |  |  |  |
|  |  | N cases / PY | HR | (95% CI) | N cases / PY | | HR | (95% CI) | Multiplicative interaction  (p-value) | RERI | (95% CI) |
|  | BMI (below median)^c^ | 14 / 3068 | 1 | (Ref.) | 22 / 4702 | | 1.09 | (0.52, 2.28) |  |  |  |
|  | BMI (above median) | 30 / 2775 | 2.03 | (0.95, 4.30) | 41 / 4130 | | 1.55 | (0.75, 3.23) | 0.45 | -0.57 | (-2.46, 1.32) |
|  |  | rs12778366 TT | | | | rs12778366 TC or CC | | |  |  |  |
|  |  | N cases / PY | HR | (95% CI) | N cases / PY | | HR | (95% CI) | Multiplicative interaction  (p-value) | RERI | (95% CI) |
|  | BMI (below median)^c^ | 28 / 5606 | 1.32 | (0.56, 3.10) | 8 / 2164 | | 1 | (Ref.) |  |  |  |
|  | BMI (above median) | 56 / 5044 | 2.30 | (0.98, 5.41) | 15 / 1860 | | 1.36 | (0.49, 3.75) | 0.66 |  | DNC |
|  |  | rs10997870 TT | | | | rs10997870 TG or GG | | |  |  |  |
|  |  | N cases / PY | HR | (95% CI) | N cases / PY | | HR | (95% CI) | Multiplicative interaction  (p-value) | RERI | (95% CI) |
|  | BMI at 20 (below median)^c^ | 11 / 2559 | 1 | (Ref.) | 21 / 3842 | | 1.14 | (0.53, 2.46) |  |  |  |
|  | BMI at 20 (above median) | 25 / 2416 | 2.32 | (1.07, 5.03) | 33 / 3800 | | 1.72 | (0.81, 3.68) | 0.37 | -0.73 | (-2.97, 1.50) |
|  |  | rs12778366 TT | | | | rs12778366 TC or CC | | |  |  |  |
|  |  | N cases / PY | HR | (95% CI) | N cases / PY | | HR | (95% CI) | Multiplicative interaction  (p-value) | RERI | (95% CI) |
|  | BMI at 20 (below median)^c^ | 24 / 4569 | 1.30 | (0.55, 3.09) | 8 / 1833 | | 1 | (Ref.) |  |  |  |
|  | BMI at 20 (above median) | 46 / 4539 | 2.47 | (1.10, 5.53) | 12 / 1677 | | 1.47 | (0.55, 3.95) | 0.66 |  | DNC |
|  |  | rs10997870 TT | | | | rs10997870 TG or GG | | |  |  |  |
|  |  | N cases / PY | HR | (95% CI) | N cases / PY | | HR | (95% CI) | Multiplicative interaction  (p-value) | RERI | (95% CI) |
|  | Waist circumference (below median)^c^ | 12 / 2330 | 1.03 | (0.46, 2.33) | 18 / 3573 | | 1 | (Ref.) |  |  |  |
|  | Waist circumference (above median) | 28 / 3356 | 1.47 | (0.70, 3.06) | 42 / 5029 | | 1.19 | (0.59, 2.39) | 0.73 | 0.24 | (-1.06, 1.54) |
|  |  | rs12778366 TT | | | | rs12778366 TC or CC | | |  |  |  |
|  |  | N cases / PY | HR | (95% CI) | N cases / PY | | HR | (95% CI) | Multiplicative interaction  (p-value) | RERI | (95% CI) |
|  | Waist circumference (below median)^c^ | 21 / 4210 | 1.10 | (0.46, 2.63) | 9 / 1693 | | 1.06 | (0.39, 2.92) |  |  |  |
|  | Waist circumference (above median) | 54 / 6133 | 1.56 | (0.78, 3.12) | 16 / 2251 | | 1 | (Ref.) | 0.48 | -0.52 | (-2.66, 1.61) |
|  |  | rs10997870 TT | | | | rs10997870 TG or GG | | |  |  |  |
|  |  | N cases / PY | HR | (95% CI) | N cases / PY | | HR | (95% CI) | Multiplicative interaction  (p-value) | RERI | (95% CI) |
|  | Physical activity^a^ (medium or high)^d^ | 21 / 3367 | 1.12 | (0.63, 2.00) | 28 / 4922 | | 1 | (Ref.) |  |  |  |
|  | Physical activity^a^ (low) | 22 / 2227 | 1.55 | (0.87, 2.78) | 32 / 3638 | | 1.29 | (0.75, 2.21) | 0.86 | 0.14 | (-1.04, 1.34) |
|  |  | rs12778366 TT | | | | rs12778366 TC or CC | | |  |  |  |
|  |  | N cases / PY | HR | (95% CI) | N cases / PY | | HR | (95% CI) | Multiplicative interaction  (p-value) | RERI | (95% CI) |
|  | Physical activity^a^ (medium or high)^d^ | 39 / 6071 | 1.37 | (0.68, 2.79) | 10 / 2218 | | 1 | (Ref.) |  |  |  |
|  | Physical activity^a^ (low) | 42 / 4160 | 1.91 | (0.94, 3.91) | 12 / 1704 | | 1.16 | (0.46, 2.89) | 0.72 | 0.38 | (-1.03, 1.79) |
|  |  | rs10997870 TT | | | | rs10997870 TG or GG | | |  |  |  |
|  |  | N cases / PY | HR | (95% CI) | N cases / PY | | HR | (95% CI) | Multiplicative interaction  (p-value) | RERI | (95% CI) |
|  | Early life energy restriction^b^ (yes)^e^ | 6 / 1464 | 1 | (Ref.) | 13 / 2079 | | 2.16 | (0.58, 8.02) |  |  |  |
|  | Early life energy restriction^b^ (no) | 38 / 4522 | 3.08 | (0.92, 10.26) | 52 / 7023 | | 2.22 | (0.67, 7.31) | 0.13 |  | DNC |
|  |  | rs12778366 TT | | | | rs12778366 TC or CC | | |  |  |  |
|  |  | N cases / PY | HR | (95% CI) | N cases / PY | | HR | (95% CI) | Multiplicative interaction  (p-value) | RERI | (95% CI) |
|  | Early life energy restriction^b^ (yes)^e^ | 15 / 2546 |  |  | 4 / 998 | |  | |  |  |  |
|  | Early life energy restriction^b^ (no) | 69 / 8370 |  |  | 21 / 3175 | | data not shown due to <5 cases | | data not shown due to <5 cases |  | DNC |
| CIMP- CRC |  | rs10997870 TT | | | | rs10997870 TG or GG | | |  |  |  |
|  |  | N cases / PY | HR | (95% CI) | N cases / PY | | HR | (95% CI) | Multiplicative interaction  (p-value) | RERI | (95% CI) |
|  | BMI (below median)^c^ | 43 / 3116 | 1 | (Ref.) | 88 / 4749 | | 1.34 | (0.88, 2.04) |  |  |  |
|  | BMI (above median) | 57 / 2822 | 1.51 | (0.94, 2.40) | 86 / 4221 | | 1.34 | (0.87, 2.06) | 0.16 | -0.51 | (-1.39, 0.37) |
|  |  | rs12778366 TT | | | | rs12778366 TC or CC | | |  |  |  |
|  |  | N cases / PY | HR | (95% CI) | N cases / PY | | HR | (95% CI) | Multiplicative interaction  (p-value) | RERI | (95% CI) |
|  | BMI (below median)^c^ | 90 / 5681 | 1 | (Ref.) | 41 / 2183 | | 1.02 | (0.66, 1.59) |  |  |  |
|  | BMI (above median) | 104 / 5154 | 1.20 | (0.85, 1.67) | 39 / 1890 | | 1.10 | (0.70, 1.73) | 0.75 | -0.11 | (-0.81, 0.59) |
|  |  | rs10997870 TT | | | | rs10997870 TG or GG | | |  |  |  |
|  |  | N cases / PY | HR | (95% CI) | N cases / PY | | HR | (95% CI) | Multiplicative interaction  (p-value) | RERI | (95% CI) |
|  | BMI at 20 (below median)^c^ | 56 / 2605 | 1.84 | (1.14, 2.97) | 77 / 3885 | | 1.62 | (1.10, 2.54) |  |  |  |
|  | BMI at 20 (above median) | 32 / 2457 | 1 | (Ref.) | 73 / 3874 | | 1.42 | (0.91, 2.23) | 0.11 | -0.64 | (-1.64, 0.37) |
|  |  | rs12778366 TT | | | | rs12778366 TC or CC | | |  |  |  |
|  |  | N cases / PY | HR | (95% CI) | N cases / PY | | HR | (95% CI) | Multiplicative interaction  (p-value) | RERI | (95% CI) |
|  | BMI at 20 (below median)^c^ | 93 / 4640 | 1.43 | (0.89, 2.30) | 40 / 1850 | | 1.44 | (0.84, 2.48) |  |  |  |
|  | BMI at 20 (above median) | 79 / 4625 | 1.08 | (0.67, 1.74) | 26 / 1706 | | 1 | (Ref.) | 0.78 | -0.10 | (-0.95, 0.76) |
|  |  | rs10997870 TT | | | | rs10997870 TG or GG | | |  |  |  |
|  |  | N cases / PY | HR | (95% CI) | N cases / PY | | HR | (95% CI) | Multiplicative interaction  (p-value) | RERI | (95% CI) |
|  | Waist circumference (below median)^c^ | 30 / 2367 | 1 | (Ref.) | 63 / 3616 | | 1.48 | (0.91, 2.43) |  |  |  |
|  | Waist circumference (above median) | 67 / 3415 | 1.38 | (0.82, 2.33) | 106 / 5121 | | 1.25 | (0.75, 2.08) | 0.11 | -0.62 | (-1.62, 0.39) |
|  |  | rs12778366 TT | | | | rs12778366 TC or CC | | |  |  |  |
|  |  | N cases / PY | HR | (95% CI) | N cases / PY | | HR | (95% CI) | Multiplicative interaction  (p-value) | RERI | (95% CI) |
|  | Waist circumference (below median)^c^ | 66 / 4275 | 1.04 | (0.63, 1.72) | 27 / 1708 | | 1 | (Ref.) |  |  |  |
|  | Waist circumference (above median) | 124 / 6250 | 1.05 | (0.64, 1.71) | 49 / 2286 | | 1.01 | (0.57, 1.79) | 0.99 | -0.01 | (-0.79, 0.78) |
|  |  | rs10997870 TT | | | | rs10997870 TG or GG | | |  |  |  |
|  |  | N cases / PY | HR | (95% CI) | N cases / PY | | HR | (95% CI) | Multiplicative interaction  (p-value) | RERI | (95% CI) |
|  | Physical activity^a^ (medium or high)^d^ | 54 / 3403 | 1 | (Ref.) | 93 / 4998 | | 1.14 | (0.80, 1.64) |  |  |  |
|  | Physical activity^a^ (low) | 45 / 2284 | 1.04 | (0.67, 1.61) | 80 / 3697 | | 1.16 | (0.79, 1.71) | 0.94 | -0.02 | (-0.61, 0.58) |
|  |  | rs12778366 TT | | | | rs12778366 TC or CC | | |  |  |  |
|  |  | N cases / PY | HR | (95% CI) | N cases / PY | | HR | (95% CI) | Multiplicative interaction  (p-value) | RERI | (95% CI) |
|  | Physical activity^a^ (medium or high)^d^ | 103 / 6154 | 1 | (Ref.) | 44 / 2247 | | 1.18 | (0.80, 1.72) |  |  |  |
|  | Physical activity^a^ (low) | 90 / 4263 | 1.08 | (0.78, 1.49) | 35 / 1718 | | 1.07 | (0.70, 1.64) | 0.56 | -0.18 | (-0.86, 0.49) |
|  |  | rs10997870 TT | | | | rs10997870 TG or GG | | |  |  |  |
|  |  | N cases / PY | HR | (95% CI) | N cases / PY | | HR | (95% CI) | Multiplicative interaction  (p-value) | RERI | (95% CI) |
|  | Early life energy restriction^b^ (yes)^e^ | 21 / 1484 | 1 | (Ref.) | 39 / 2112 | | 1.29 | (0.72, 2.32) |  |  |  |
|  | Early life energy restriction^b^ (no) | 81 / 4604 | 1.34 | (0.79, 2.28) | 140 / 7133 | | 1.46 | (0.88, 2.42) | 0.61 | -0.18 | (-1.10, 0.75) |
|  |  | rs12778366 TT | | | | rs12778366 TC or CC | | |  |  |  |
|  |  | N cases / PY | HR | (95% CI) | N cases / PY | | HR | (95% CI) | Multiplicative interaction  (p-value) | RERI | (95% CI) |
|  | Early life energy restriction^b^ (yes)^e^ | 43 / 2580 | 1 | (Ref.) | 17 / 1015 | | 1.15 | (0.62, 2.12) |  |  |  |
|  | Early life energy restriction^b^ (no) | 156 / 8530 | 1.23 | (0.84, 1.80) | 65 / 3208 | | 1.32 | (0.86, 2.03) | 0.84 | -0.07 | (-0.89, 0.76) |
| Abbreviations: CRC, colorectal cancer; CI, confidence interval; DNC, did not converge; HR, hazard ratio; MSS, microsatellite stable; N, number of; PY, person-years at risk; Ref., reference; RERI, relative excess risk due to interaction.  ^a^ Low versus moderate or high physical activity was <8 versus ≥8 kJ/min of occupational energy expenditure in men and ≤30 versus >30 min/day of non-occupational physical activity in women.  ^b^ Exposure status was based on the place of residence during the Dutch Hunger Winter, i.e. lived in a Western city versus lived in a Western rural area or non-western area.  ^c^ Composite SIRT1 genotype and body composition models [separate models for waist circumference (trouser/skirt size adjusted for BMI (kg/m2)) and BMI and BMI at 20 (median split categories)] included age, sex, physical activity (median split), diet [total energy intake (kcal/day), alcohol intake (binary split, ≥30 g/day), consumption of red meat, and consumption of vegetables and grains (g/day)], first-degree family history of CRC (yes/no) and smoking status (never, ex, current) as potential confounders.  ^d^ Composite SIRT1 genotype and physical activity (occupational for men and non-occupational for women) model included age, sex, BMI, diet (as above), first-degree family history of CRC and smoking status as potential confounders.  ^e^ Composite SIRT1 genotype and energy restriction model included age, sex, BMI, physical activity, diet (as above), first-degree family history of CRC and smoking as potential confounders | | | | | | | | | | | |
